# Acupuncture for diabetic neurogenic bladder: A protocol for systematic review and meta-analysis

**DOI:** 10.1097/MD.0000000000024573

**Published:** 2021-02-12

**Authors:** Yu Dai, Qing Ye, Yi-ming Sun, Xin-ru Liu, Lu Li, Quan Wen, Tian-min Zhu

**Affiliations:** a Chengdu Eighth People's Hospital; b Chengdu University of Traditional Chinese Medicine, Chengdu, Sichuan Province, China.

**Keywords:** acupuncture, diabetic neurogenic bladder, meta-analysis, systematic review

## Abstract

**Background:**

Diabetic neurogenic bladder (DNB) is one of the common complications of diabetes mellitus, which has a high prevalence rate. Some research suggested that acupuncture can improve the clinical symptoms of diabetic neurogenic bladder patients, but there is no systematic review or meta-analysis to assess this therapy. Therefore, this study aims to explore the effectiveness and safety of acupuncture for patients with DNB.

**Methods:**

In this study, we will search for electronic databases including the Cochrane Library, Web of Science, PubMed, MEDLINE, EMBASE,

China National Knowledge Infrastructure (CNKI), Wan-Fang, and Baidu Scholar Database from inception to December 2020. We will select randomized controlled trials that have been published in English or Chinese related to acupuncture for DNB. Selection of study, extraction of data, and assessment of study quality will be performed independently by 2 researchers, and we will use Revman 5.3 software which is provided by Cochrane assistance network, to perform the data analysis.

**Results:**

This study will provide evidence of the effectiveness and safety of acupuncture for DNB.

**Conclusion:**

This study will clarify whether acupuncture is an effective treatment for DNB, and will also provide a reference for clinical practice and guidelines development.

## 1 Introduction

Diabetes mellitus is a common endocrine and metabolic disorder disease, which is characterized by chronic hyperglycemia. Five hundred seventy-eight million people are expected to be suffered from diabetes by 2030.^[1]^ Diabetes not only has a high prevalence rate but also has a variety of acute and chronic complications. Diabetic neurogenic bladder (DNB) or diabetic bladder dysfunction (DBD) is one of the common complications of diabetes mellitus,^[2]^ which has a high prevalence rate, and a study has shown that diabetic patients have a 40% to 80% chance of developing DNB.^[3]^ Even under good blood glucose control, DNB will still occurs in 25% of diabetic patients.^[3,4]^ In addition, the longer the history of diabetes, the higher the probability of DNB.^[5]^ The pathogenesis of DNB is still unclear, which may be related to the dysfunction of autonomic nerves (especially parasympathetic nerves) that is caused by long-term hyperglycemia, and it leads to abnormal urination reflex and bladder dysfunction, then giving rise to frequency, urgency, nocturia, urinary incontinence, and so on.^[6,7]^ If not treated properly, patients can repeatedly develop a urinary tract infection, leading to renal function damage or even progression to renal failure.^[8]^

Western medicine treatment of DNB is based on the control of blood glucose, and then give the corresponding drug treatment and non-drug treatment,^[3,9,10]^ still, these treatments have low expectations and high consumption, which will increase the burden of family and society over time.

Acupuncture is a traditional Chinese medicine treatment. Acupuncturists select specific points to treat diseases according to traditional Chinese medicine theory.^[11]^ Moreover, it can be widely used for its high curative effect, low side effect, and only requires simple operations. Some research suggested that acupuncture can improve the clinical symptoms of diabetic neurogenic bladder patients.^[12–16]^ Although acupuncture can help treat DNB, there is no systematic review or meta-analysis to investigate its safety and effectiveness. Therefore, it is necessary to carry out a systematic review of the literature concerning the safety and efficacy of acupuncture for treatment of DNB.

## 2 Method

### 2.1 Study registration

This protocol has been registered on INPLASY: the registration number is INPLASY2020120076. The protocol report is based on the Preferred Reporting Items for Systematic Reviews and Meta-Analyses Protocols (PRISMA-P) statement guidelines.^[17]^

### 2.2 Inclusion criteria

#### 2.2.1 Types of participants

Participants who are over the age of 18 years diagnosed as diabetes with bladder dysfunction will be included. All participants will be included regardless of education, and economic position, age, gender, and race.

#### 2.2.2 Types of study

Randomized controlled trials and quasi-randomized controlled trials of acupuncture for DNB will be included. Case report, animal studies, meta-analysis, reviews, conference articles will be excluded.

#### 2.2.3 Types of interventions and comparisons

Simple acupuncture or acupuncture combined with other conventional therapy (such as Chinese herbal, western medicine) used to manage patients with DNB will be included in the observation group. The control group was treated with other conventional treatment and not undergoing any acupuncture therapy (as auricular acupuncture, electroacupuncture, warm acupuncture, acupoint injection, acupoint catgut embedding, etc)

#### 2.2.4 Types of outcomes

The primary outcome is the total effective rate.

Secondary outcomes include urodynamic parameters (such as daily micturition frequency, residual bladder volume, maximum urine flow rate) and adverse events.

### 2.3 Search strategy

We will search electronic databases including the Cochrane Library, Web of Science, PubMed, MEDLINE, EMBASE, China National Knowledge Infrastructure (CNKI), Wan-Fang, and Baidu Scholar Database. All databases will be searched from inception to December 2020, and the language of the study should be English or Chinese.

The search terms will include “diabetic neurogenic bladder,” “Diabetic bladder dysfunction,” “diabetic cystopathy,” “acupuncture,” ”acupuncture therapy,” ”needing,” ”acupoint,” ”electroacupuncture.” The sample of search strategy on PubMed is summarized in Table 1.

**Table 1:** Search strategy for PubMed.

Number	Search terms
1	Diabetic neurogenic bladder
2	Diabetic bladder dysfunction
3	Neurogenic bladder, diabetic
4	Diabetic cystopathy
5	Cystopathy, Diabetic
6	Or 1–5
7	Acupuncture
8	Acupuncture therapy
9	Needing
10	Acupoint
11	Electroacupuncture
12	Or 7-11
13	Randomized controlled trial
14	Controlled trial
15	Clinical trial
16	Randomly
17	Random
18	Or 13–17
19	6 and 12 and 18

### 2.4 Data collection and analysis

#### 2.4.1 Selection of studies

**Figure 1:**
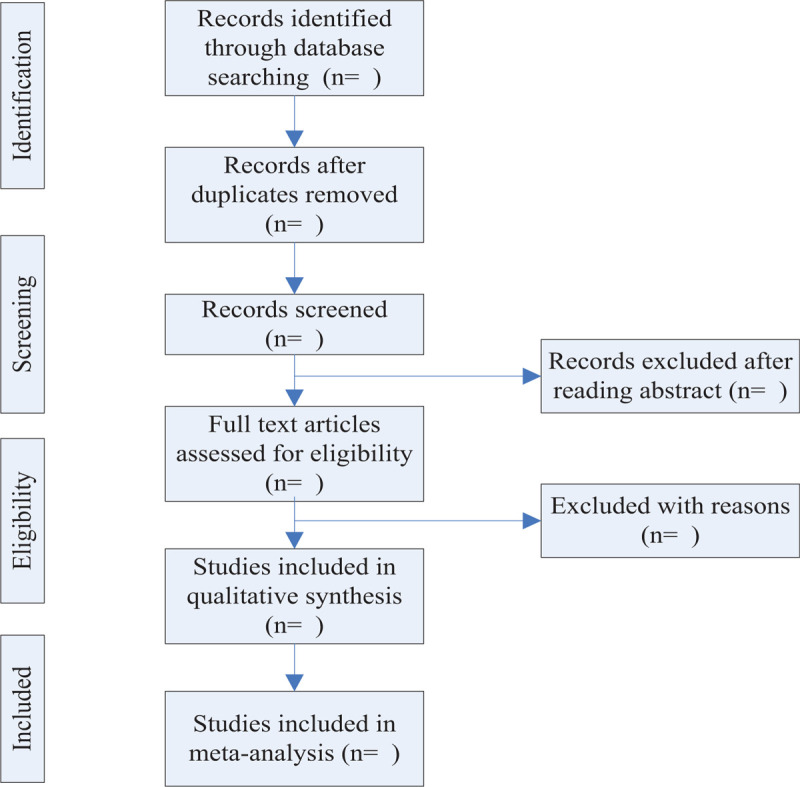
PRISMA flow chart.

All searched literature will be imported into the Endnote software 7.0 for management and deduplication. Two reviewers will scan all retrieved literature's title and abstract in strict line with the inclusion and exclusion criteria independently, then exclude the unqualified literature. After that, the reviewers will read the full-text of all potential studies to determine final inclusions according to the full criteria. If there are doubts and differences, the 2 reviewers will solve it through discussion. We will use a PRISMA flow chart to show the process of research selection (Fig. 1).

#### 2.4.2 Data extraction and management

Two authors of our team will extract data from the qualified articles. The extracted data include first author, year of publication, participants’ demographic data (such as gender, age), sample size, study design, study duration, interventions, outcome measures, main results, follow-up, and other information. We will use Microsoft Excel 2013 software to establish a data extraction table, and the extracted details will be recorded in it. If there are any discrepancies in the process, the third author will join in the discussion and consultation.

#### 2.4.3 Assessment of risk of bias

The Cochrane risk of bias tool will be used to assess 7 aspects of risk of bias, including sequence generation, allocation concealment, blinding of participants and assessors, blinding of outcome assessment, incomplete outcome data, selective reporting, and other bias, which can evaluate the risk of bias of the final included studies. We will divide the risk of bias into 3 levels: low risk, high risk, and unclear. This assessment will be conducted independently by 2 reviewers, and any differences in the assessment process will be resolved through consultation with the third reviewer.

#### 2.4.4 Measures of treatment effect

We will use Revman 5.3 software which is provided by Cochrane assistance network to perform data analysis. For continuous outcomes, we will use mean difference (MD) with 95% confidence intervals (CIs) to measure the treatment effect, and risk ratio (RR) with 95% CIs will be used to analyzed dichotomous data.

#### 2.4.5 Assessment of missing data

For the continuous data, if the standard deviation SD is missing, at first, we will consider contacting the author. If it fails, we can choose to carry out conversion calculation according to the formula in Cochrane handbook or borrow the data results from previous studies.

#### 2.4.6 Assessment of heterogeneity and subgroup analysis

We will use Cochrane Q-test and *I*^*2*^ index to determine heterogeneity. The random-effects model will be used if the evaluation of heterogeneity is significant (*P* ≤ .10, *I*^*2*^ *>* 50%). When the probability value *>* 0.10 or *I*^*2*^ index was lower than 50%, that means the heterogeneity is not significant, and then we will use fixed-effects model. When heterogeneity exists, if the source of heterogeneity can be determined, we will perform subgroup analysis according to heterogeneity factors; if the source of heterogeneity cannot be determined, the random-effects model will be used to merge the data.

#### 2.4.7 Sensitivity analysis

Sensitivity is an important index to measure the quality and heterogeneity of studies. Therefore, we will carry out the sensitivity analysis to access the stability and reliability of the research results.

#### 2.4.8 Publication bias

If there are more than 10 studies included, we will use funnel plot to evaluate publication bias. If the number of studies is less than 10, the power of funnel plot is too low to detect publication bias.

## 3 Discussion

The treatment of DNB is difficult, and modern medical treatment for DNB is also limited. Some systematic reviews have indicated that acupuncture is beneficial to diabetes and neurogenic bladder.^[18–21]^ Acupuncture has existed for more than 2500 years, which is an integral part of traditional Chinese medicine, commonly used in the treatment of diabetes and its complications.^[22,23]^ Besides, studies have also reported that acupuncture is useful for DNB patients. However, there is no systematic and comprehensive analysis on the therapeutic effect of acupuncture on diabetic neurogenic bladder. Therefore, we will intend to conduct a systematic review and meta-analysis on acupuncture for diabetic neurogenic bladder, to provide evidence for the effectiveness and safety of acupuncture treatment of DNB, and provide a reliable basis for clinical treatment.

## Author contributions

**Conceptualization:** Quan Wen, Tian-min Zhu

**Funding acquisition:** Quan Wen

**Investigation:** Qing Ye, Yi-ming Sun, Xin-ru Liu

**Methodology:** Yu Dai

**Writing – original draft:** Yu Dai

**Writing – review & editing:** Lu Li, Tian-min Zhu

## Abbreviations

Abbreviations: CNKI = China National Knowledge Infrastructure, DBD = diabetic bladder dysfunction, DNB = Diabectic neurogenic bladder, PRISMA = preferred reporting items for systematic reviews and meta-analyses.
